# Integrative multiomics analysis of *Premolis semirufa* caterpillar venom in the search for molecules leading to a joint disease

**DOI:** 10.1038/s41598-020-79769-y

**Published:** 2021-01-21

**Authors:** Giselle Pidde, Milton Y. Nishiyama, Ursula Castro de Oliveira, Isadora M. Villas-Boas, Adriana F. Paes-Leme, Inácio L. Junqueira-de-Azevedo, Rafael Marques-Porto, Carla C. Squaiella-Baptistão, Denise V. Tambourgi

**Affiliations:** 1 Laboratório de Imunoquímica, Instituto Butantan, São Paulo, Brazil; 2 Laboratório Especial de Toxinologia Aplicada, Center of Toxins, Immune-Response and Cell Signaling (CeTICS), Instituto Butantan, São Paulo, Brazil; 3 Laboratório Nacional de Biociências, Centro Nacional de Pesquisa Em Energia E Materiais, Campinas, São Paulo Brazil; 4 Laboratório de Desenvolvimento E Inovação, Instituto Butantan, São Paulo, Brazil

**Keywords:** Biochemistry, Biological techniques, Computational biology and bioinformatics, Immunology, Molecular biology, Diseases, Pathogenesis, Rheumatology

## Abstract

The joint disease called pararamosis is an occupational disease caused by accidental contact with bristles of the caterpillar *Premolis semirufa*. The chronic inflammatory process narrows the joint space and causes alterations in bone structure and cartilage degeneration, leading to joint stiffness. Aiming to determine the bristle components that could be responsible for this peculiar envenomation, in this work we have examined the toxin composition of the caterpillar bristles extract and compared it with the differentially expressed genes (DEGs) in synovial biopsies of patients affected with rheumatoid arthritis (RA) and osteoarthritis (OA). Among the proteins identified, 129 presented an average of 63% homology with human proteins and shared important conserved domains. Among the human homologous proteins, we identified seven DEGs upregulated in synovial biopsies from RA or OA patients using meta-analysis. This approach allowed us to suggest possible toxins from the pararama bristles that could be responsible for starting the joint disease observed in pararamosis. Moreover, the study of pararamosis, in turn, may lead to the discovery of specific pharmacological targets related to the early stages of articular diseases.

## Introduction

The Brazilian moth *Premolis semirufa* (Walker, 1856), family Erebidae, or, popularly, pararama in its larval stage, lives in the Amazon rainforest, almost exclusively on the rubber tree *Hevea brasiliensis*, feeding on its leaves. Contact with the caterpillar or cocoon bristles causes intense itching, followed by symptoms of acute inflammation such as pain, heat and redness, which resolve within hours or days^1,2^. However, a chronic inflammatory reaction, characterized by articular synovial membrane thickening with joint deformities, common to chronic synovitis, frequently develops in individuals after only one or repeated accidents^3^. This chronic inflammatory process characterizes pararamosis. As an important occupational disease with morbid consequences that tends to specifically affect rubber tappers in areas of the Brazilian State of Pará, “pararama-associated phalangeal periarthritis” was added to the “Manual of diagnosis and treatment of accidents by venomous animals” by the Brazilian Ministry of Health in 1992. So far, no effective treatment for pararamosis exists^4^.

Studies on the caterpillar and the associated envenomation are scarce, both regarding the toxic substances released by its bristles and on the molecular mechanisms involved in the resulting pathogenesis. The pararama bristles crude extract has been shown to be proteolytic and to induce an intense inflammatory process characterized by the presence of neutrophils in the paw tissues of injected mice and a strong and specific antibody response^1^. We next confirmed that the strong cellular and humoral immune responses induced by bristle components, in a murine model, consisted of an intense neutrophil and macrophage infiltration into the envenomation site^2^. Additionally, proliferation/migration and activation of T and B lymphocytes to the draining lymph nodes and elevated plasma levels of IL-6, IL-10, IL-12, IL-17, and IL-23 were observed^2^. Later, it was demonstrated that the bristles’ extract activates the human complement system, generating biologically active fragments, such as C3a, C4a, and C5a anaphylatoxins, and that it contains a serine protease, named Ps82, with proteolytic activities similar to those observed for the whole extract^5^.

Despite our efforts to characterize the pararama’s bristle toxins, the molecules triggering the process leading to articular damages in pararamosis are still unknown. In the absence of specific treatments directed to specific toxins, pararamosis is symptomatically treated based on other similar joint diseases, such as osteoarthritis (OA) and rheumatoid arthritis (RA)^3,4^.

Loss of articular cartilage structure and function is among the hallmarks of OA^6^. The gradual articular cartilage degradation, bone and anatomical changes, low-grade inflammation and synovitis associated with pain, and swelling, reduce mobility and quality of life^7^. Similar degenerative changes are observed in RA affected joints, with inflammatory cells present in the synovial fluid. Though the etiology of RA is not fully known, several factors, alone or in combination, can lead to its development. Genetics, autoimmunity, smoking and microbial infections may increase the incidence of RA^8^. This disease is characterized by macrophages, granulocytes, and B lymphocytes infiltrating the synovial tissues, which thicken and invade the bone, cartilage, and tendons, causing pain and disrupting joint functions^9^. Lysosomal enzymes released by granulocytes and macrophages in the synovium destroys bones. These degenerative changes disrupt joint functions and cause fibrous and bone ankyloses^10^.

Identification and validation of biochemical markers for OA and RA have been sought after to predict disease development in subjects at risk, improve diagnosis at an earlier stage and distinguish subgroups and phenotypes. Additionally, known biomarkers can help monitor disease progression, assess treatment responses and outcomes and identify therapeutic targets^11,12^. Transcriptome data are valuable resources to finely analyze putatively important protein sequences and find homologies in model species or other well-studied organisms. Therefore, articular diseases may provide a basis for the identification of possible pararama toxins involved in pararamosis.

We used a multi-omics approach to determine the bristle components involved in pararamosis. The pararama transcriptomic and proteomic profiles were analyzed in search of putative toxins and were compared to a public RNA-seq study of synovial biopsies. Our results on this species’ genetics are unprecedented and our approach allowed us to identify pararama bristle components possibly involved in triggering the joint disease pararamosis.

## Material and methods

### Caterpillar

Pararama caterpillar specimens were collected in a rubber tree plantation area, around S 01° 08.201′ W 047° 45.844′ Alt. 39 m, in São Francisco do Pará, Brazil. The license for capture, transport and maintenance of the animals (protocol number: 11971-2) was given by the Chico Mendes Institute for Biodiversity Conservation (ICMBIO) of the Brazilian Ministry for the Environment. The animals were maintained at the Immunochemistry Laboratory, Instituto Butantan, SP, Brazil. The access to pararama biological material was authorized by the Brazilian Institute of Environment and Renewable Natural Resources (IBAMA) (process number: 02001.008743/2011-12, authorization 01/2009) and by the National System of Genetic Heritage Management and Associated Traditional Knowledge (SisGen) (registration: A05C092, 05/11/2018), both agencies of the Brazilian Ministry of the Environment.

### Library preparation and sequencing for transcriptomic analysis

Since pararama venom glands consist of microscopic structures distributed along its dorsal subepithelial tissue^13,14^, samples of dorsal integument obtained from ten caterpillar specimens were used for the transcriptome sequencing. To avoid contamination with material from the bristles, they were removed with forceps, and the dorsal integument (about 2 mm deep) was cut off with scissors. TRIzol Reagent (Invitrogen, Carlsbad, CA, USA) was added to the tissue for the extraction of total RNA, according to the manufacturer’s protocol. The integrity of the RNA was evaluated with an Agilent 2100 Bioanalyzer RNA nanochip series (Agilent Technologies Inc., Santa Clara, CA, US). The polyA mRNAs were selected with oligo dT bound to magnetic beads using the Dynabeads mRNA DIRECT kit (Invitrogen, Life Technologies Corp., Carlsbad, CA, US). The mRNA was quantified using the Quant-iT RiboGreen RNA reagent and Kit (Invitrogen, Life Technologies Corp.). The mRNA was used to prepare the complementary DNA (cDNA) library with the TruSeq Stranded mRNA Library Preparation Kit (Illumina, San Diego, CA), according to the manufacturer’s instructions. Briefly, the mRNA was fragmented by heating at 94 °C in fragmentation buffer. Double-stranded cDNA was synthesized, end-repaired and A-tailed. Sequencing adapters were then linked to the cDNA fragments, according to the manufacturer’s protocol. The cDNAs solution was enriched over 15 cycles of PCR amplification. The quality of the library was evaluated by size distribution of the cDNA libraries measured by a 2100 Bioanalyzer with DNA1000 assay (Agilent Technologies), showing an average insert size of 160 bp (without adapters). An ABI StepOnePlus Real-Time PCR System was used in quantification of the sample library before sequencing. The cDNA library was sequenced using the Illumina HiSeq 1500 System, into a Rapid paired-end flow cell in a 300 cycles of 2*150 bp paired-end strategy.

### Transcriptome raw data processing

The BCL image files conversion and demultiplexing were conducted using the Illumina Casava software (version: 1.8.2), with Illumina quality control Q30, for generating a pair of paired-end “fastq” files. RNA-Seq raw data reads were filtered for the detection of PhiX contaminant, using the software bowtie2 version 2.2.5^15^. The raw sequencing reads were pre-processed through an “*in house*” pipeline for sequencing quality control, to trim and remove reads with low-complexity and homopolymer enriched regions, poly-A/T/N tails, the adapter sequences and low-quality bases with the software fastq-mcf 1.04.662^16^. The reads were trimmed if the mean quality score was lower than 25 in a window size equal to 15 and filtered out if more than 90% of reads corresponded to homopolymers or low-complexity regions, or smaller than 40 bp. The raw data generated in this project was deposited in the NCBI BioProject section under the accession code PRJNA382571 and BioSample SAMN06710513. This Transcriptome Shotgun Assembly was deposited at NCBI TSA under the accession GFNN00000000.

### Pararama transcriptome assembly

To generate a non-redundant set of transcripts, we performed a de novo assembly of the transcriptome by Trinity software^17^ using 41,483,084 RNA-seq good quality paired-reads, with parameter CuffFly to reduce the number of false-positive isoforms. The minimum transcript length in the assembly was set to 300 bp.

### Transcriptome abundance estimation

The putative ribosomal genes were filtered out from assembled transcripts for the expression profile analysis, using as reference the Metazoa rRNA database, 2016^18^, and BLASTn alignment with cut-offs of e-value < 1e^−20^, query coverage > 60% and query identity > 70%. In order to estimate transcript abundance, the paired-end reads were aligned back with the pararama assembled transcriptome. Further computing of the abundance for each transcript was performed by RSEM^19^, along with a Maximum Likelihood abundance estimates, using the Expectation–Maximization algorithm for its statistical model. Final abundance estimates were calculated as Fragments Per Kilobase of exons per Million fragments mapped (FPKM) and Transcripts Per Million (TPM) values.

### Transcriptome functional annotation

The pararama transcriptome was annotated using the BLASTx^20^ alignment tool with a cut-off *e*-value < 1e^−5^ against multiple protein databases, such as the Gene Ontology (GO)^21^, the NCBI Transcriptome Shotgun Assembly (TSA_NR) and UniProt-Swissprot^22^.

For the prediction of Open Reading Frames (ORFs), we used the TransDecoder software (http://transdecoder.sourceforge.net/) in the assembled transcripts, considering only predicted proteins with a protein length ≥ 60aa. The signal peptides were predicted using the software SignalP 4.0^23^. The predicted amino acid sequences were aligned by BLASTp against the above protein databases. The analysis of retained PFAM domains for the proteins annotation was performed with the *hmmsearch* tool in the software package^24^, against a PFAM protein families database^25^, with a cut-off e-value < 1e^−3^. A priority order of UniPro/Swissprot, PFAM database and NR-NCBI protein database hits was used for the annotation and selection of the best candidate for each transcript.

The transcriptome annotation for KEGG pathways, KEGG orthology (KO) and enzyme classification was assigned using the online KEGG Automatic Annotation Server (KAAS, http://www.genome.jp/tools/kaas/). ^26^ The analyses were conducted based on the Bi-directional Best Hit (BBH) method with BHRs Score >  = 60 to obtain KEGG Orthologs (KO) assignment, and using as reference, two closely related species of *Ixodes scapularis* and *Bombyx mori* from Arthropoda phylum available in the KEGG database.

The protein homologs between *P. semirufa* and *H. sapiens* were searched for conserved domains, using the CD-search tool^27^, a more reliable approach, composed by a reference database of NCBI-curated domains and data imported from Pfam (Protein Families), SMART (Simple Modular Architecture Research Tool), COG (Clusters of Orthologous Groups of proteins), PRK (Protein K(c)lusters, and TIGRFAMs (The Institute for Genomic Research's database of protein families) using the cut-off e-value ⩽ 1e−3.

### Protein isolation and mass spectrometry analysis

The bristles were cut with scissors separating them from the integument, and later processed as previously standardized^1^. The bristles were suspended in cold phosphate-buffered saline–PBS (8.1 mM sodium phosphate, 1.5 mM potassium phosphate, 137 mM sodium chloride and 2.7 mM potassium chloride, pH 7.2). The solution was frozen and then macerated with the aid of a glass stick, homogenized and centrifuged at 5606×*g* for 20 min at 4 °C. This procedure was done twice. The supernatant was collected and its protein content was determined by using the BCA Protein Assay Kit (Pierce Biotechnology, MA, USA). Supernatant aliquots were stored at − 80 °C until use. The extract proteins (100 µg of protein) were denatured using 1.6 M urea, reduced with 5 mM dithiothreitol and alkylated with 14 mM iodoacetamide for 30 min at room temperature and subjected to in-solution trypsin digestion (20 ng/µL). The samples were then acidified to pH 3.0 with 5% TFA. The acidified digest was desalted and concentrated using Sep-Pak C18 columns (Waters) according to the manufacturer's instructions. Peptides (4.5 μl, ~ 2 μg) were separated by RP-nanoUPLC (nanoAcquity, Waters) using a C18 (100 μm × 100 mm) column, under a 2–90% acetonitrile gradient in 0.1% formic acid over 45 min, at a flow rate of 0.6 μl/min. The chromatographer was coupled to a ESI-Q-Tof Premier mass spectrometer (Waters) and analyzed in both MS and MS–MS modes^28^.

### Mass spectrometry data analysis

We used MassLynx v.4.1 to analyze the acquired spectra. The data, converted to peak using Mascot, were then searched against the list of predicted proteins from the pararama transcriptome (14,956 proteins from 21,259 transcripts) using Mascot engine v.2.3.01 (Matrix Science Ltd.) as described in Aragão et al. (2012). Peptides with a minimum of five residues with significant threshold (p < 0.05) were retained. The pararama proteome and transcriptome data were integrated in order to predict protein functional annotation and to identify putative toxins.

### Phylogenetic analysis

Putative serine protease sequences were selected to perform protein evolution analysis with the software Prottest 2.4^29^, which uses the best-fit model of evolution, among a set of candidate models, for a given protein sequence alignment; with fixed relative rates of amino acid replacement matrix (WAG^30^), with site heterogeneity model gamma (WAG + G). The Bayesian analyses were performed using Markov chain Monte Carlo (MCMC) implemented in BEAST 1.8 package^18^, with nine categories to gamma, a lognormal uncorrelated clock and a speciation model following the birth and death model. In addition, we ran four independent MCMC searches using distinct randomly generated starting trees, consisting of 50-million generations each of them, with the trees sampled every 1,000 generations. Convergence was inspected in Tracer v1.6 (http://tree.bio.ed.ac.uk/software/tracer). All runs reached a stationary level after 10% BurnIn with a large effective sample size (ESS > 5000). Trees obtained after the BurnIn step were used to generate a maximum clade credibility tree with TreeAnnotator v1.8.2^18^, using a majority rule. The resulting tree was visualized and edited using FigTree v1.4.0 (unpublished, available at http://tree.bio.ed.ac.uk/software/figtree).

### Network and pathway analysis

In order to investigate the biological pathways interactions triggered by pararama envenomation, we used the MetaCore (Clarivate Analytics), which analyses the predicted toxin proteins and delineates functional gene networks. Therefore, we used as targets the 220 unique proteins identified in the proteomic analysis of pararama bristles. The protein sequences were aligned against the *Homo sapiens* protein sequences, by using the BLASTx alignment tool with the threshold of p-value ⩽ 1e−9 and CD-Search tool for conserved domains with the threshold of e-value ⩽ 1e−3, and 129 homologous genes were identified.

The networks were constructed using the algorithm "network analysis". We applied the shortest paths algorithm to establish directed paths between the selected objects, following the main parameters: (1) relative enrichment with the uploaded data (the 129 human-homologous proteins in this study), and (2) relative saturation of networks with canonical pathways. To determine a statistically significant biological process or pathway, the threshold of FDR < 0.05 was adopted.

The Enriched Reactome pathways were identified using Cytoscape ClueGO^31^, with a Kappa Score Threshold = 0.4. To compute the enriched pathways and to correct the p-value, a two-sided hypergeometric (Enrichment/ Depletion) test and Benjamini–Hochberg correction were applied, respectively, and the Enriched pathways were considered as significant if p-value < 0.05.

### RNA-seq study of synovial biopsies

In order to determine the existence of bristles extract components that could be responsible for triggering the inflammatory process that results from this peculiar envenomation, we crossed the DEGs obtained from synovial tissues of osteoarthritis (OA) and rheumatoid arthritis (RA) patients, representative of the upregulated genes, with the identified proteins in the proteome of pararama bristles. The transcriptome datasets of patients with RA, OA and healthy samples were downloaded from the Gene Expression Omnibus (GEO) under the accession code GSE89408. The statistical tests of the RNA-seq data were performed in R with the limma package^32^. Counts were converted to log2 counts per million, quantile normalized, and precision weighted. A linear model was fitted to each gene, and empirical Bayes-moderated t statistics were used to assess differences in expression^33^. The RA patient samples and, similarly, the OA patient samples were grouped and considered as replicates for the identification of DEGs between the RA patients’ group, the OA patients’ group and the healthy controls. The analyses were conducted using the edgeR package with adjusted p-value ≤ 0.001 and fold-change ≥ 3.

## Results

### Pararama multi-omics data analysis

**Figure 1:**
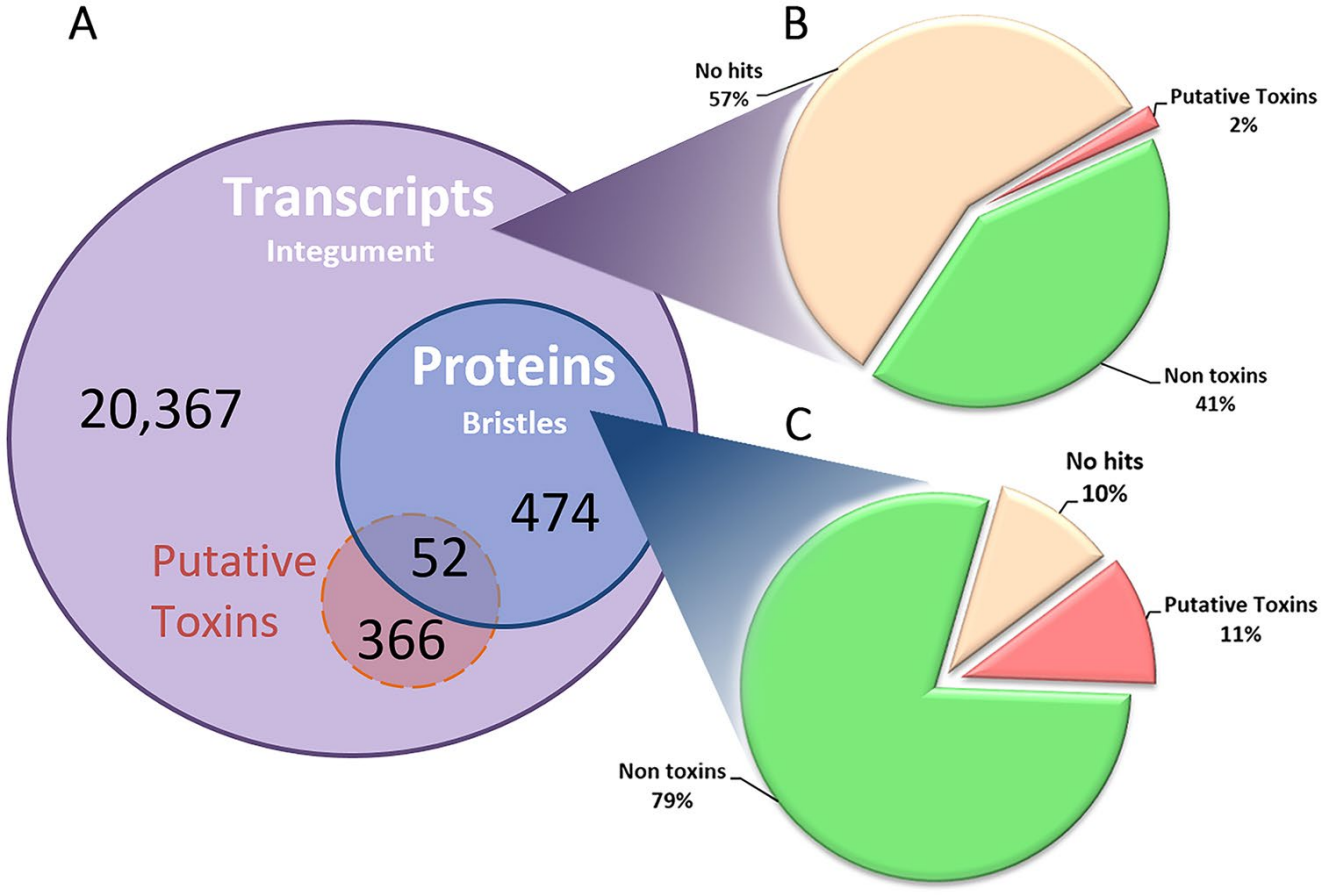
Global and integrative transcriptomic and proteomic analyses. (**A**) Venn diagram showing a general overview of the transcripts in the integument of pararama caterpillar, identified by NGS, and bristle proteins by mass spectrometry. The transcriptome contained 21,256 transcripts (purple), among which 418 have similarity to known toxins (red). Proteomic analysis was performed using the transcriptome database, and 526 transcripts were found (blue), among which 52 showed similarity with known toxins. (**B**) Pie chart showing the number of transcripts in the integument, among which 57% were no hits, 41% were non-toxins and 2% were putative toxins. (**C**) Pie chart showing proteins from the bristles extract, among which 10% were no hits, 79% were non-toxins and 11% were putative toxins. The figure was drawn using Microsoft Excel version 1.5 (www.microsoft.com/).

Figure S1 shows an overview of the data analysis strategy used in this study. Sequencing of the transcriptome of pararama integument resulted in a total of 86,834,040 reads. After removing reads containing adaptor sequences, short reads and low-quality reads from the raw sequence data, 84,284,372 good reads remained (97.06% of the total) (Table 1). De novo assembly using Trinity was performed on good reads generating 21,259 transcripts (Fig. 1A), 19,062 transcripts with an N50 value of 919 bp, a median length of 519 bp and an average transcript length of 749 bp (Table 1). The raw and assembled transcripts data are available at NCBI under BioProject ID PRJNA382571.

**Table 1:** Summary of the pararama integument transcriptome.

Total number of reads	86,834,040
Total good reads	84,284,372
Total number of transcripts	21,259
Total number of unigenes (trinity 'genes')	19,062
Total assembled bases	15,878,674 bp
Median Transcript Length	519 bp
Average Transcript Length	749 bp

For identification through proteomic analysis, the bristle proteins were analyzed by LC–MS/MS, and after automatic peptide-spectrum matching, against the predicted proteins from the transcriptome of the integument of pararama, 526 proteins (220 unique proteins) were identified (Fig. 1A and Supplementary Table S1).

In order to identify and differentiate the toxin and non-toxin proteins in the pararama transcriptome and proteome, the predicted proteins were aligned against several databases, such as Uniprot, Animal Toxin Database, TSA, PFAM and Gene Ontology. In the annotation process of the transcriptome from the caterpillar integument, we identified 9145 transcripts (43%), corresponding to 41% non-toxins and 2% putative toxins. 12,114 transcripts were classified as unknown products, accounting for 57% of the transcripts (Fig. 1B).

The proteomic analysis of the bristles extract revealed 472 known proteins, which correspond to 79% non-toxins and 11% putative toxins. Unidentified proteins accounted for 10% (54 proteins) (Fig. 1C).

### Functional analysis of predicted proteins

**Figure 2:**
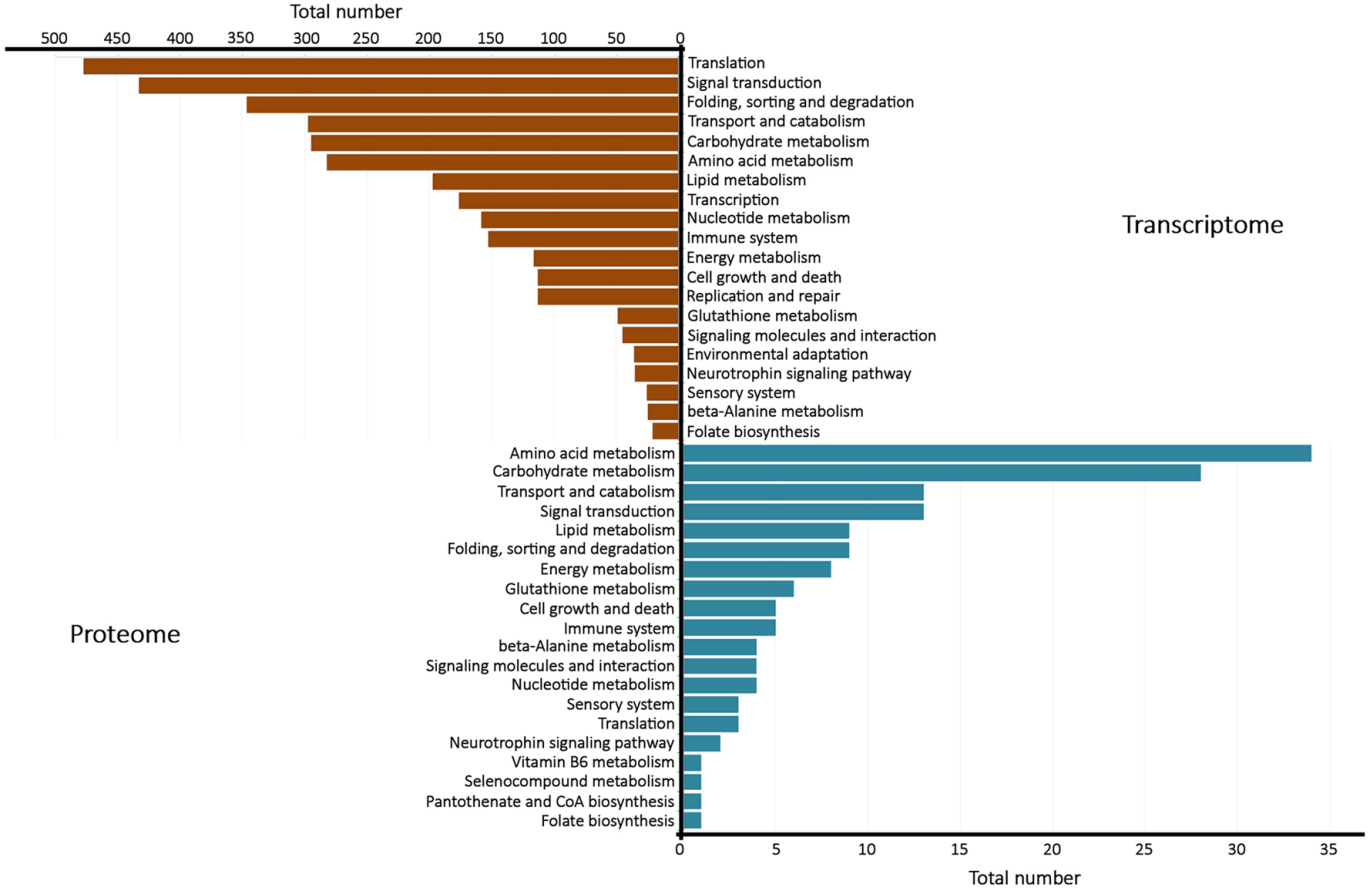
Enriched pathways. Top 20 enriched KEGG pathways among the annotated transcripts in the transcriptome and the proteome. The total number of transcripts were 5,022 and 251 for the transcriptome and the proteome, respectively. The pathways were grouped manually in more general terms. The figure was drawn using Microsoft Excel version 1.5 (www.microsoft.com/).

KEGG annotation of the assembled transcripts was performed using the KAAS software^26^. A total of 5022 (23.62%) assembled transcripts were assigned to KEGG Pathways and with KEGG Ortholog (KO) identifiers in the transcriptomic and 251 (47.7%) in the proteomic analyses. 3547 unique KOs were identified by integrating the transcriptome and proteome data. Figure 2 presents the major functional groups classified into KEGG Pathways. The major pathways, both in the transcriptome (14.11%) and in the proteome (14.74%), are associated with metabolic pathways.

The most representative pathways in the transcriptome are Metabolic pathways (comprised by 709 transcripts), Ribosome (192 transcripts), Spliceosome (121 transcripts), Protein processing in endoplasmic reticulum (119 transcripts) and RNA transport (119 transcripts). The five main pathways obtained from the proteome analysis were Metabolic pathways (37 molecules), Carbon metabolism (14 molecules), Biosynthesis of amino acids (12 molecules), Cysteine and Methionine metabolism (7 molecules) and Oxidative phosphorylation (6 molecules) (Fig. 2).

**Figure 3:**
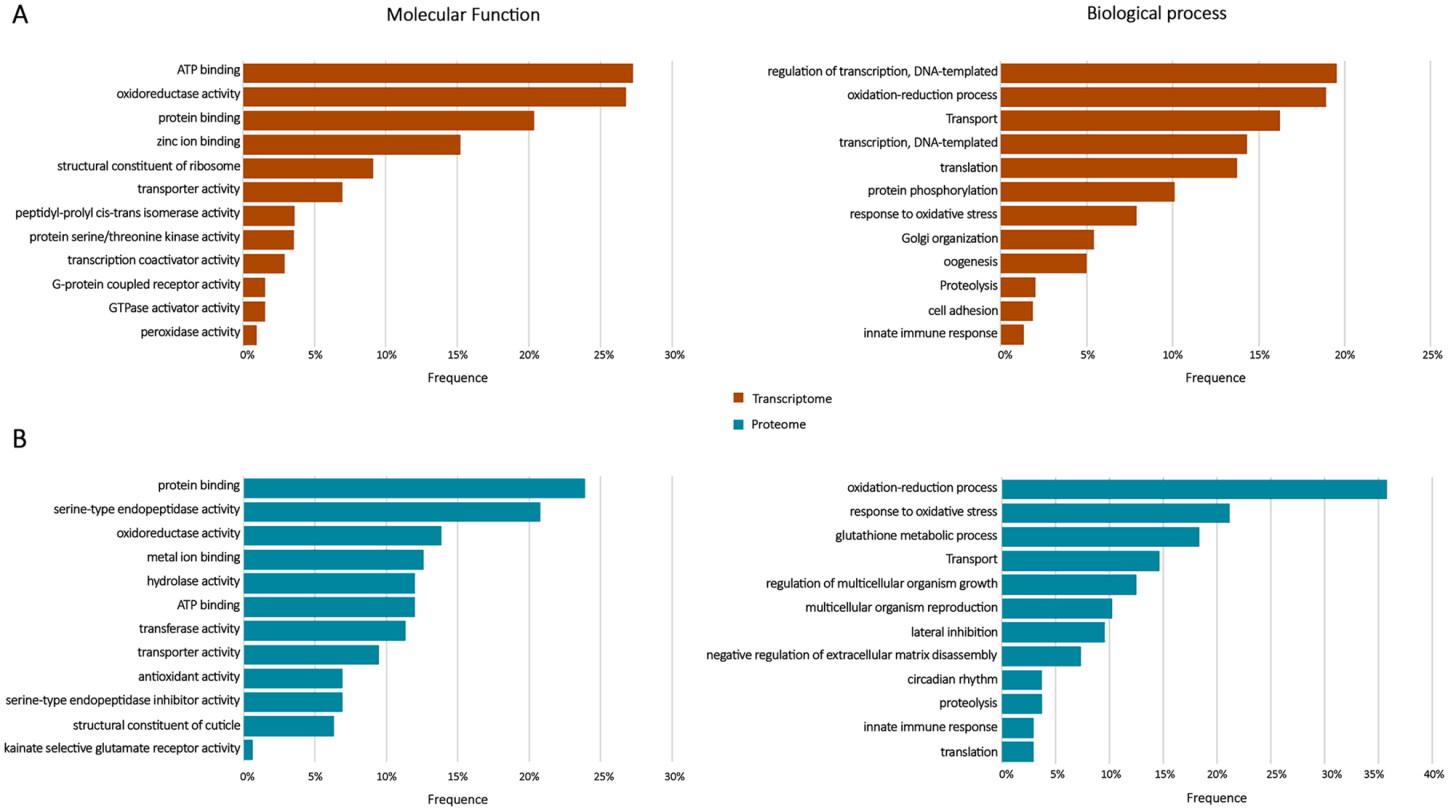
Functional classifications analysis. The Gene Ontology enrichment analysis was performed based on unique sequence homology. (**A**) Transcripts from the integument of pararama and (**B**) bristle proteins were classified into two main categories: molecular function and biological process, each one including 12 functional groups. The figure was drawn using Microsoft Excel version 1.5 (www.microsoft.com/).

Annotation of the non-toxin transcripts using the Gene Ontology (GO) terms resulted in 7235 transcripts in the transcriptome and 124 predicted proteins in the proteome, mapped into two categories: Molecular function and Biological process. Figure 3 shows the most abundant GO‐term categories found in the transcriptomic and proteomic analyses. The numbers of mapped GO terms for molecular functions and biological processes were 7780 and 7281 in the transcriptome, and 159 and 137 in the proteome, respectively.

The most abundant Molecular Function ontologies in the transcriptome were binding (3791 transcripts, 48.7%) and catalytic activity (2588 transcripts, 33.3%). ATP binding, oxidoreductase activity, protein binding, zinc ion binding and structural constituent of ribosome are the five most enriched, containing 27.2%, 26.7%, 20.3%, 15.2% and 9.0%, respectively. Concerning Biological Processes, Cellular processes (1423 transcripts, 19.5%) and Metabolic processes (1375 transcripts, 18.9%) were the most highly represented in the transcriptome, of which the top five were transcription regulation, DNA-templated (19.5%), oxidation–reduction processes (18.8%), Transport (16.2%), transcription, DNA-templated (14.3%) and protein phosphorylation (10%) (Fig. 3A).

In the proteome, the most abundant Molecular Function ontologies were catalytic activity (83 proteins, 52.2%) and binding (64 proteins, 40.2%). The main ones were protein binding (23.9%), serine-type endopeptidase activity (20.8%), oxidoreductase activity (13.8%), metal ion binding (12.6%) and hydrolase activity (11.9%). Oxidation–reduction processes, response to oxidative stress, glutathione metabolic processes, transport and regulation of multicellular organism growth were the five most enriched Biological Processes in the proteome, containing 35.8%, 21.2%, 18.2%, 14.6% and 10.2%, respectively (Fig. 3B).

### Major enzymes and metabolic pathways in pararama

**Figure 4:**
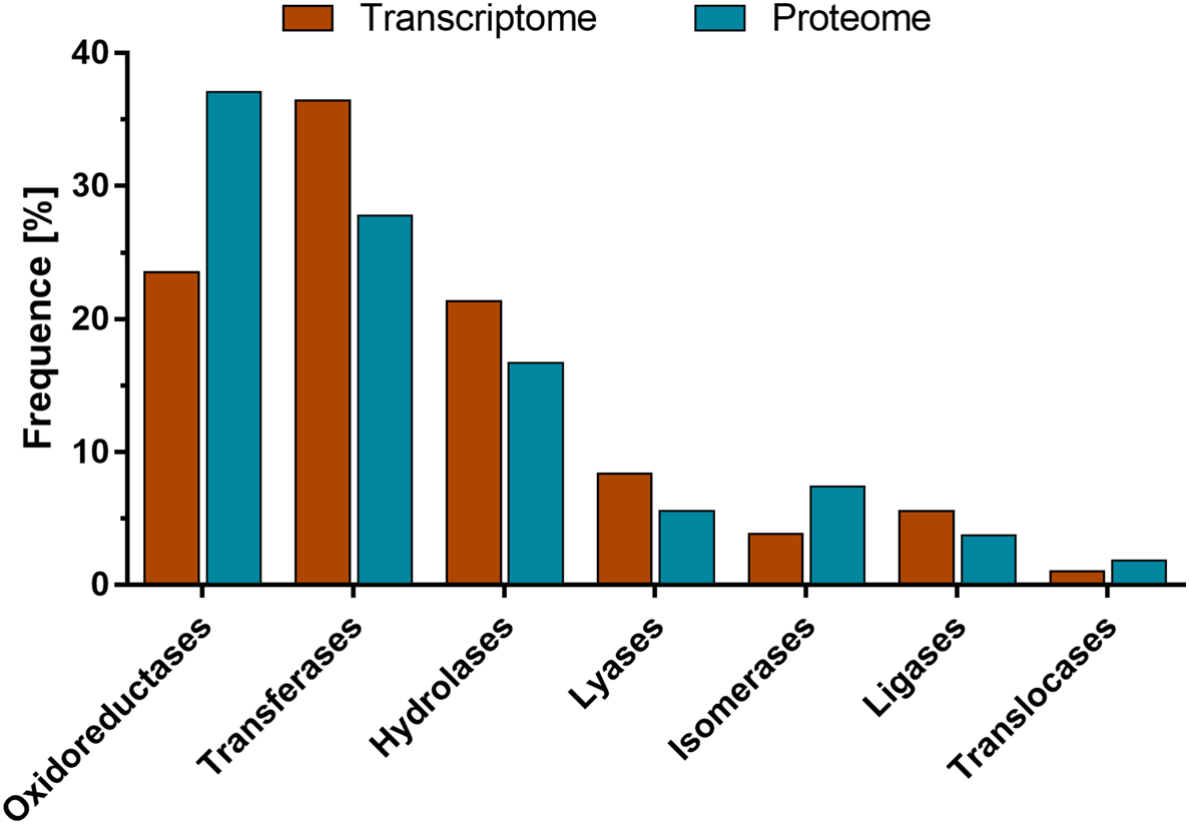
Enzymes distribution. Frequency of molecules identified using the KEGG databases for the pararama transcriptome and proteome. The figure was drawn using GraphPad Prism version 7.0 (www.graphpad.com/).

The categorization and identification of the transcripts in the basis of Enzyme Commission (EC) Numbers were established through KEGG Orthology assignments. A total of 2324 transcripts were assigned to 1689 unique KOs and 596 ECs. Enzyme classification revealed that transferases constitute the largest group among pararama enzymes (36.4%, 217 enzymes), followed by oxidoreductases (23.5%, 140 enzymes), hydrolases (21.3%, 127 enzymes), lyases (8.4%, 50 enzymes), ligases (5.5%, 33 enzymes), isomerases (3.8%, 23 enzymes) and translocases (1%, 6 enzymes) (Fig. 4).

Similarities were found in the annotation of the proteome with 73 proteins, corresponding to 65 unique KOs, with a total of 54 unique enzymes. The most abundant enzymes were oxidoreductases (37%, 20 enzymes), followed by transferases (27.7%, 15 enzymes), hydrolases (16.6%, 9 enzymes), isomerases (7.4%, 4 enzymes), lyases (5.5%, 3 enzymes), ligases (3.7%, 2 enzymes) and translocases (1.8%, 1 enzyme) (Fig. 4).

### Identification of putative toxins

**Figure 5:**
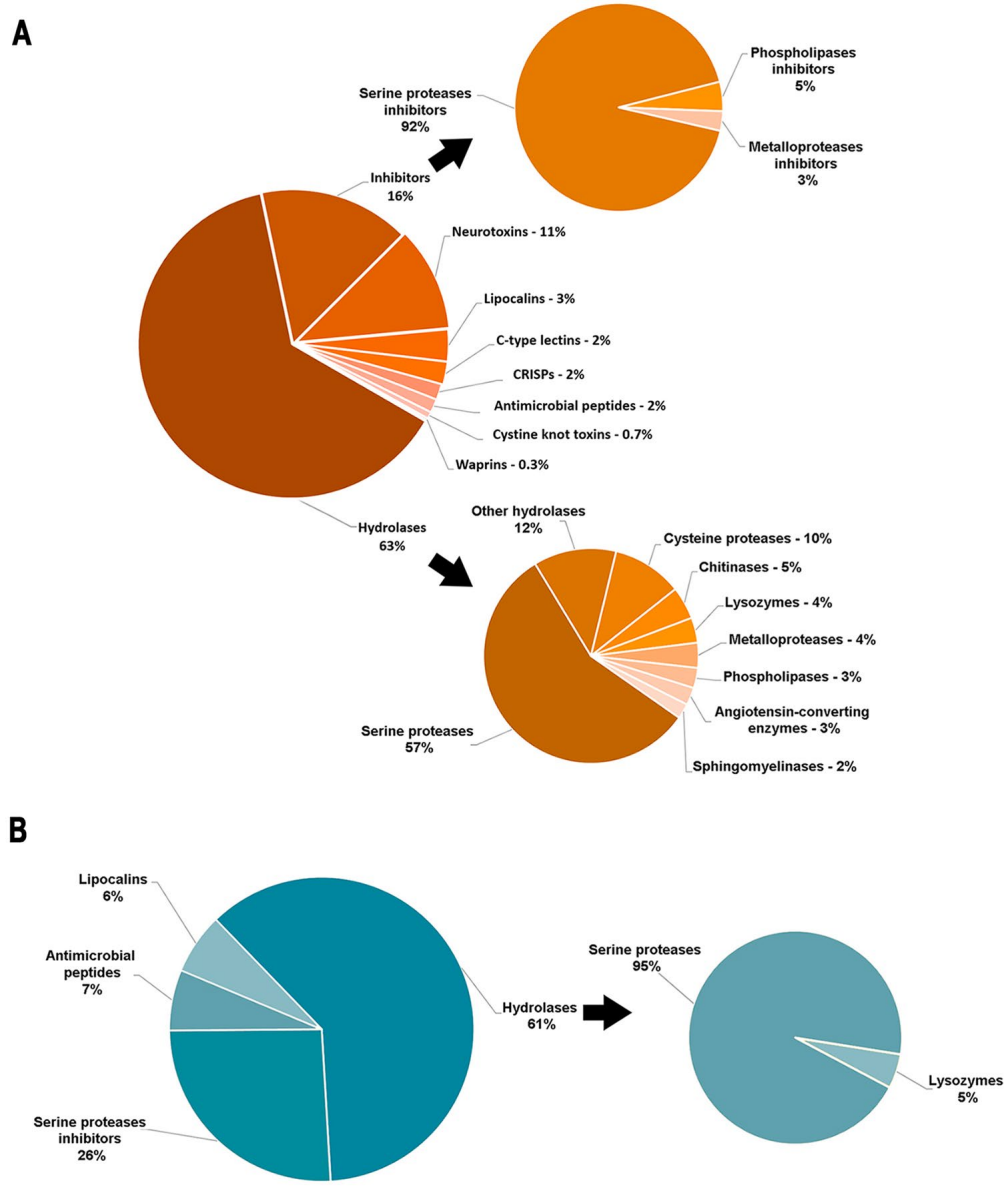
Putative toxins. Classification of putative toxins in the pararama (**A**) integument and (**B**) bristles. The relative abundance of proteins from different toxin families is shown in this pie chart. Major putative toxins from pararama are serine proteases and their inhibitors. The figure was drawn using Microsoft Excel version 1.5 (www.microsoft.com/).

In this study, we used different strategies to identify putative toxins. After combining and curating the annotation, we identified 418 transcripts (2%) for putative toxins in the transcriptome, *i.e.*, those matching known toxin transcripts. The analysis of putative toxins revealed that hydrolases (63%) are the dominant class. Inhibitors (16%) and neurotoxins (11%) are also abundant components, whereas proteins belonging to the lipocalins (3%), C-type lectins (3%), Cysteine-Rich Secretory Proteins—CRISPs (2%), antimicrobial peptides (2%), cystine knot toxins (0.7%) and waprins (0.3%) were in minor proportions. Hydrolases are composed mainly of serine proteases (57%), followed by cysteine proteases (10%), chitinases (5%), lysozymes (4%), metalloproteases (4%), phospholipases (3%), angiotensin-converting enzymes (3%), sphingomyelinases (2%) and 12% of other hydrolases, such as esterases and lipases. Inhibitors are predominantly (92%) composed of serine protease inhibitors. We also identified phospholipase (5%) and metalloprotease inhibitors (3%) (Fig. 5A).

In the proteome, 31 protein sequences showed similarity to known toxin sequences. Hydrolases were also the dominant proteases (61%), 95% of which were serine proteases and 5% lysozymes. Serine protease inhibitors constitute the second most abundant proteins in the pararama proteome (26%). Antimicrobial peptides and lipocalins were also found, at 7% and 6%, respectively (Fig. 5B).

To assess gene expression levels, we determined the FPKM and TPM values for each transcript (Supplementary Table S2). The most expressed transcripts of the putative toxins are antimicrobial peptides, a first line of defense against infectious microorganisms. Forty-eight percent of these putative toxins were identified in the transcriptome of the integument and correspond to 66% of the proteins identified in the bristles (Supplementary Figure S2).

### Phylogenetic analysis of pararama serine proteinases

Considering the proteolytic character of pararama’s venom^1,5^ and the common occurrence of serine proteinases in several animal venoms, we further investigated the diversity of these enzymes within our data. Using combined transcriptomic and proteomic analyses, we detected 25 proteins from the pararama bristles extract showing significant similarity to serine proteinases, containing the typical peptidase S1 superfamily conserved domain. In order to better understand the evolution of these proteins and their possible role in the particular envenomation process caused by pararama, we compared the eight most abundant sequences identified in the proteomic analysis with different serine proteinase clans from various Lepidoptera species. Supplementary Figure S3A presents a multiple sequence alignment of the most abundant serine proteinase domains from the proteomic analysis with those from other Lepidopterans. The classic catalytic triad (His, Asp, Ser) is indicated with a “*#*”. The phylogenetic tree of these proteases is shown in Supplementary Figure S3B. The peptidase S1 superfamily splits into four distinct branches, though we classified them in three clans: snake venom-like serine protease (2 proteins), serine protease (3 proteins) and trypsin like (3 proteins).

### Pathways mapping and envenomation process

We used the Metacore and Reactome platforms to analyze the highly interconnected gene networks and to determine the pathways, in the context of regulatory networks, and molecules that may act in the envenomation process. The analyses were performed based on pararama molecules identified through the proteome analysis that were homologous to proteins encoded by *H. sapiens* genes. Using the BlastX alignment tool with a minimum Identity cutoff of 25%, though the mean and median were 49% and 53% identity, respectively. To improve the search for homologs, we used the %positives, in the context of the BlastX alignment, that preserves the physico-chemical properties of the original residues. In the later case, the mean and median %positives were 63% and 68, respectively (Supplementary Figure S4).

Of the 222 proteins identified in the proteome analysis, 129 presented an average of 63% homology based on identity and conserved substitutions with human proteins and more than 90% shared conserved domains with *H. sapiens* proteins. Of these 129 proteins, 25 formed an interaction network related to molecules involved in chemotaxis, cell adhesion, apoptosis and survival, cytoskeleton and ECM remodeling. All these processes are involved in the inflammatory response associated with pararamosis (Supplementary Figure S5).

**Figure 6:**
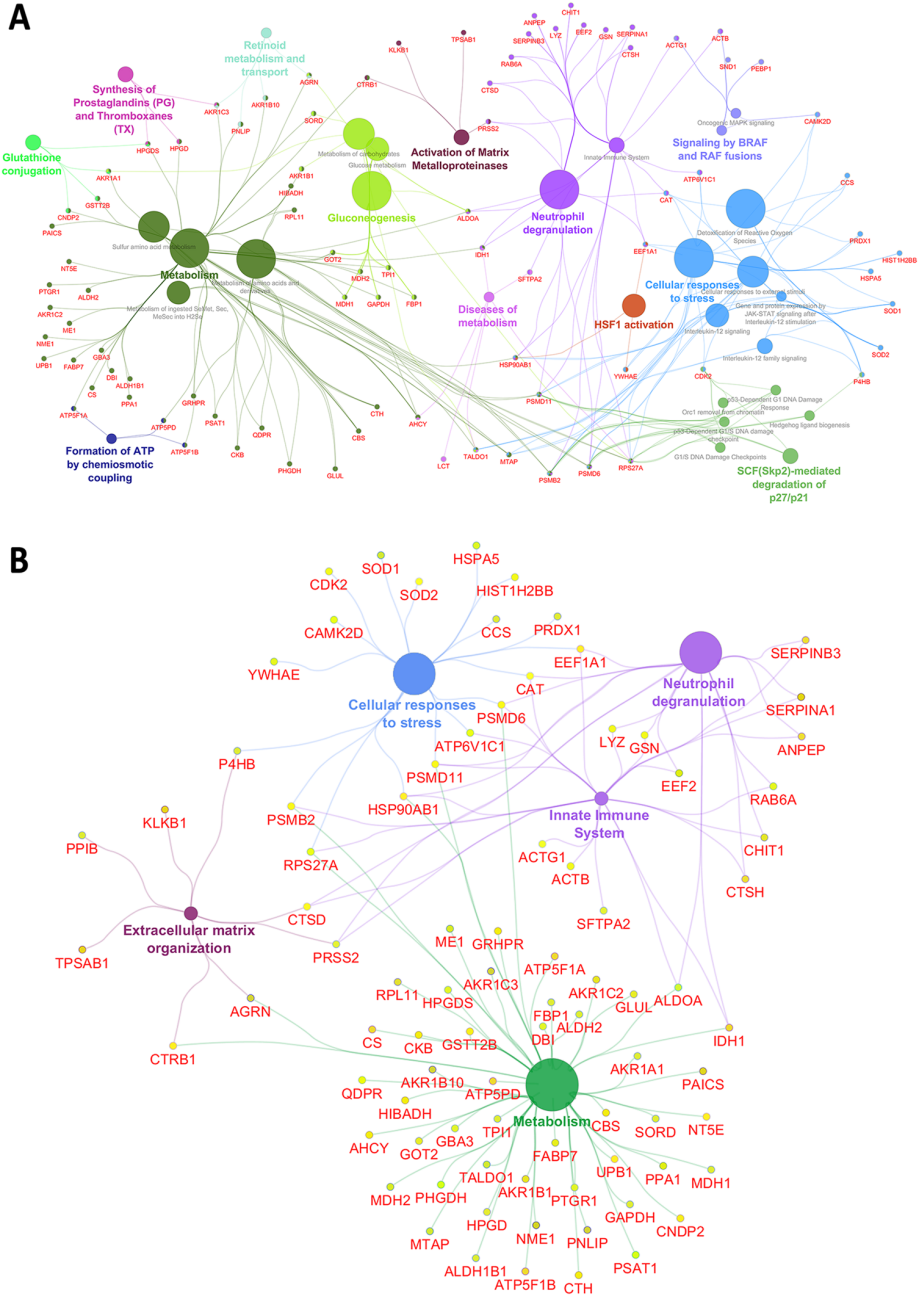
Functional enrichment in the pararama proteome. Enrichment for Reactome groups was performed using the ClueGO plugin and Cytoscape software. Nodes were coloured according to groups of related functions by association of statistically significant (FDR q-value) terms. (**A**) The general analysis revealed a complex network of 129 pararama proteins that presented an average of 63% homology based on conserved substitutions with human proteins and more than 90% of shared conserved domains, enriched in more than 30 Reactome terms. (**B**) Among them, about 80 proteins were distributed in functions that we considered relevant for the study of probable targets related to the development of pararamosis, including metabolism, extracellular matrix organization, cellular response to stress, innate immunity and neutrophil degranulation. The figure was drawn using Cytoscape ClueGO version 3.7.2 (www.cytoscape.org/).

The proteins from the pararama bristles proteome were submitted to a functional enrichment analysis. To accomplish that, we queried the Reactome annotations database using the ClueGO plugin into Cytoscape. The general analysis revealed a complex network of 129 pararama proteins enriched in more than 30 Reactome pathways (Fig. 6A). Among them, about 80 proteins were distributed in functions that we consider relevant for the development of pararamosis. The analysis revealed five enriched pathways and their associated proteins. Eighteen proteins were associated with cellular responses to stress, 24 with the innate immune system, 18 with neutrophil degranulation, 55 with metabolism and 8 with extracellular matrix organization (Fig. 6B). Most of these pathways are involved in the initial response of the organism to threats, triggering an inflammatory response, which could potentially lead to acute and/or chronic pathologies.

### Pararama bristle proteins potentially associated with joint diseases

**Figure 7:**
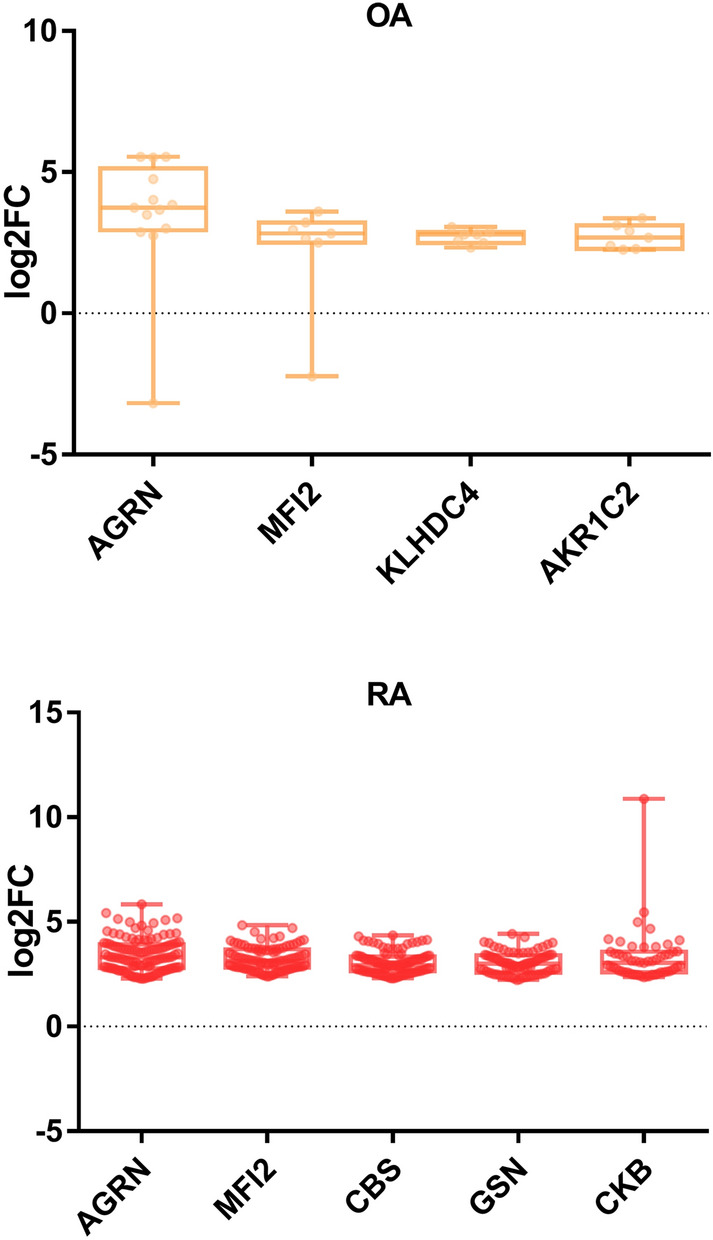
Proteins associated with joint diseases. Publicly available data (GSE89408) from human patients with osteoarthritis (OA) and rheumatoid arthritis (RA) indicating upregulated proteins were crossed with the data from the pararama bristles proteome. Seven proteins presented homology with upregulated proteins. The figure was drawn using GraphPad Prism version 7.0 (www.graphpad.com/).

The genome-wide transcriptional differences between normal healthy synovium and early RA and OA have been explored and more than 5,000 differentially expressed genes have been identified^33,34^. We crossed the DEGs from osteoarthritis and rheumatoid arthritis representative of the upregulated genes with the identified proteins in the proteome of pararama bristles. We identified seven homologous molecules differentially expressed, two upregulated in both OA and RA, agrin (AGRN) and melanotransferrin (MFI2). Kelch Domain Containing 4 (KLHDC4) and Aldo–Keto Reductase Family 1 Member C2 (AKR1C2) were exclusive to OA. The Cystathionine Beta-Synthase (CBS), Gelsolin (GSN) and Creatine Kinase B (CKB) molecules were unique to RA (Fig. 7). These seven homologous proteins were validated in silico, with more than three shared conserved domains and at least one key PFAM domain (Supplementary Tables S3 and S4).

## Discussion

The occupational joint disease known as pararamosis affects rural workers in the northern region of Brazil and has been studied by our group for the last 10 years^1,2,5^. Its etiological agents are toxic components present in the bristles of the larval form of the moth *Premolis semirufa,* or pararama, which induce local articular synovial membrane thickening with joint deformities after one or multiple contacts. We showed that the pararama bristles toxic extract is highly proteolytic and induces an intense inflammatory process, as well as strong cellular and humoral immune responses^1,2^. However, the bristle components responsible for this peculiar envenomation remained unknown. To identify putative toxins and possible biological and molecular interactions that could lead to this particular clinical picture, we have performed a global transcriptomic characterization of the glandular tissue associated with a proteomic analysis of the pararama bristles.

First, we analyzed the pararama transcriptomic and proteomic data in order to understand the venom complexity. The scarcity of Lepidoptera annotations in the databases made it difficult to identify most transcripts obtained from the integument of pararama, totaling 12,114 unknown transcripts. Even so, we obtained 9145 identified transcripts, among which 8727 were non-toxins and 418 were classified as putative toxins. The difficulty in characterizing specific transcripts from *P. semirufa* reinforces the relevance and novelty of this study.

Analyzing the data from the proteome against the transcriptomic database, we identified 415 non-toxin proteins and 57 putative toxins. The higher number of non-toxin proteins in our analysis reveals the high complexity of the samples. Unlike other venomous arthropods, such as spiders and scorpions, the pararama venom gland is a microscopic structure^13,14^, virtually impossible to isolate, which forced us to work with integument samples. Obviously, the integument also includes the transcripts of tissues adjacent to the venom gland, contributing to the large number of genes classified as non-toxins. Similarly, the sample used for the proteomic analysis was not a collected secreted venom, but a bristles extract, which certainly includes structural proteins from the bristles. On the other hand, it is important to acknowledge that patients have contact not only with the venom secreted by the gland, but also with the entire structure of the bristles. Therefore, we cannot rule out the possibility that structural bristle proteins are also involved in the pathogenesis of pararamosis. Besides, non-structural proteins that were not identified as toxins in our analysis could actually be toxins in pararama. For these reasons, some of our analysis also included the non-toxins, aiming to identify their molecular functions in the integument and/or in the bristles, as well as to investigate possible roles in pararamosis.

To detect the enriched functional terms of different protein‐gene regulation types, we quantitatively analyzed the data sets for two enrichment types: the KEGG pathways and GO categories. We identified some overlapping pathways/annotations between the transcriptome and the proteome, however in different proportions.

The most representative KEGG pathways in the transcriptomic study related to Metabolism (mainly carbohydrate metabolism) and Genetic Information Processing, and the enriched pathways in the proteomic study are more directed towards amino acid metabolism, followed by Environmental Information Processing pathways. The presumed GO biological processes corroborate KEGG results in such a way that the transcripts are mainly involved in metabolic and cellular processes involved in cell maintenance and organism survival. Differently, the GO biological processes detected in the proteome are mainly related to metabolic and cellular processes and response to stimulus. This last term may suggest a relationship with the organism defence function, including venom production.

According to their putative molecular functions, the transcripts were mainly associated with binding, followed by catalytic and transporter activities. On the other hand, the analysis of the proteome functional classification revealed that catalytic activity and binding were the main molecular functions. The most evident catalytic activity was serine-type endopeptidase activity. As observed in other animals, venoms are generally rich in proteins with enzymatic activity. Thus, our proteomic data suggest that proteins present in the bristles are, in fact, closer to typical venom composition.

Enzymes are central molecules in most activities in the body and are widely known to play important roles in venoms. The enzymatic profile shows a prevalence of the classes of oxidoreductases, transferases and hydrolases, both in the transcriptome and proteome analyses. These profiles fall within normal cellular function, but are also suggestive of higher levels of trafficking and secretion proteins. We would like to draw attention to the hydrolase class, in which the transcriptomic analysis showed a prevalence of esterases (EC 3.1.-), while the proteomic profile showed a prevalence of peptidases (EC 3.4.-).

The high-throughput-omics analyses are robust strategy for the study of venom profiles. However, these approaches have their limitations, such as the inability to detect classes or families of new toxins. Despite these problems, sequence homology-based searches of known toxins against transcriptomic and proteomic data are common strategies for the identification of putative toxins, especially in organisms from which venom is difficult to obtain. Our investigation on the repertoire of pararama toxins revealed a wide prevalence of proteases of the hydrolases class, as well as inhibitors, neurotoxins, lipocalins, C-type lectins, CRISPs, antimicrobial peptides, cystine knot toxins and wrapins.

Wrapins are small, cysteine-rich peptides members of the group of the defensins. These peptides have antibacterial and antifungal activity, and can also act as protease inhibitors^35^. The cystine knot toxins are ligands for varied ion channels. These proteins are characterized by a structural motif containing three disulfide bridges. This family exhibits toxic properties, including haemolytic, antibacterial and antiviral activities^36^. Antimicrobial peptides (AMPs) are mostly found in insects. In their majority, insect AMPs are small (20–50 residues), acting mainly against bacteria and/or fungi but also several parasites and viruses. Though their main function is immunological, these peptides can affect other aspects of vertebrate physiology^37–39^. CRISPs are widely distributed among animal venoms, such as snakes, scorpions, spiders and stingrays. Although, to date, most CRISPs have no known specific functions, they do exhibit a number of different pharmacological activities, including blockage of cyclic nucleotide-gated and voltage-gated ion channels and inhibition of smooth muscle contraction^40^. C-type lectins represent a large family of proteins with a calcium-dependent carbohydrate-binding capacity, acting in both mammalian and insect innate immunity. They are currently classified as opsonins in a large number of insects and share significant similarity in their primary structures with C-type lectins from other animals. Snake venom C-type lectins encompass a group of hemorrhagic toxins, which interfere with hemostasis^41,42^.

Arthropod lipocalins present a wide molecular and functional diversity. They act on retinol transport, invertebrate cryptic coloration, pheromone transport and prostaglandin synthesis. Lipocalins were also implicated in cellular homeostasis and in the modulation of the immune response^43,44^. Neurotoxins target ion channels and receptors in membranes of excitable cells and can act in neuromuscular transmission. These toxins can interfere with neurotransmitters, such as acetylcholine, adrenaline, dopamine, GABA, norepinephrine and γ-aminobutyrate^45^. Serine proteinase inhibitors of the Kazal, Kunitz, α-macroglobulin and serpin families have been identified and characterized in arthropod hemolymph. These hemolymph proteinase inhibitors probably act in the defense system of arthropods against pathogens or parasites and in the regulation of physiological processes. However, these proteins have also been intensively studied for their actions in human plasma, since they are important regulators of serine proteinases involved in inflammation, blood clotting, fibrinolysis and complement activation processes^46,47^.

Proteases constitute a vital portion of venoms. Serine proteases are involved in a number of physiological processes and are present in all living organisms. They mediate a wide range of biological functions, including pathological, inflammatory and haemostatic processes. In insects, several serine proteases take part in coagulation and in activation cascades, such as the complement system, in response to wounds and infections^48,49^. Previously, we have shown^5^ that a serine protease from the whole pararama caterpillar bristles extract activates the human complement system, which may contribute to the inflammatory process following envenomation in humans. The majority of the putative toxins identified are serine proteases of different types. To understand the distribution of their putative functions, we performed a phylogenetic analysis using the most expressed representative toxins. Our analysis revealed serine proteases form specific clusters in the phylogenetic tree, which we classify into three distinct groups, snake like serine proteases, serine proteases and trypsin like proteases, suggesting specific and targeted activities in the physiology of the caterpillar^50^. This result also highlights the presence of snake-like serine proteases, known for a number of biological functions in the envenomation process, such as interfering with the coagulation cascade^51^.

Altogether, our conventional analyses of the pararama transcriptomic and proteomic data resulted in a small number of putative toxins and proteins traditionally related to animal venom composition. On the other hand, patients do not have contact only with the venom secreted by the gland, but with the entire structure of the bristles, and we cannot exclude the possibility that proteins not classified as toxins are also related to the pathogenesis of pararamosis. We introduced an innovative approach in our workflow in order to speculate on the possible pathogenic functions of pararama proteins regardless of whether they are classified as toxins or not. This approach consisted of the homology analysis of pararama bristle proteins with *Homo sapiens* proteins, aiming to identify pararama proteins that are not classified as toxins, but that could take part in human physiological processes, such as joint diseases and, by association, in pararamosis.

The idea consisted in identifying bristle proteins that present similarity to human proteins and that might induce inflammatory effects, like those caused by the analogous human protein when deregulated. Several examples of this kind of similarity have been described in animal venoms, such as the cobra venom factor (CVF) found in Elapidic venoms^52^ and the thrombin-like enzymes (SVTLEs), found in several snake venoms, such as Bothro*ps* sp and *Crotalus* sp^53^. CVF mimics human C3 and binds to the same target, Factor B, activating the complement system^54^. SVTLEs exert the same fibrinogen cleaving activity as thrombin^55^. Deprived of endogenous regulation, these activities lead to severe pathologies. In this work, we expanded the search for similarities in an approach that may be useful in the study of other venoms.

The 129 proteins present in the pararama bristles extract that are homologous to human proteins were submitted to a pathway enrichment analysis, using the Metacore and Reactome platforms, and also compared to a public RNA-seq study of synovial biopsies from patients with osteoarthritis and rheumatoid arthritis. Performing a network analysis in Metacore, we found 25 human-like pararama proteins that could initiate multiple signaling pathways involved in the regulation of inflammatory responses^56,57^. This network leads to the identification of the hypothetical production of molecules related to the processes of chemotaxis^58^, cell adhesion^59^, apoptosis and survival^60^, cytoskeleton remodeling and ECM remodeling^61^, associated with joint diseases. Among the hypothetical products of this network, CCL2, IL-6 and IL-8 have been detected in chondrocyte cultures stimulated with the pararama bristles extract^62^.

The Reactome analysis revealed a complex network enriched in more than 30 pathways. Among them, about eighty proteins were distributed in functions that we consider relevant for the development of pararamosis, including metabolism^63^, extracellular matrix organization^64^, cellular response to stress^65^, innate immunity^66^ and neutrophil degranulation^67^. These are processes that have been widely implicated in the pathogenesis of other joint diseases, such as osteoarthritis and rheumatoid arthritis.

Most striking is the identification of eighteen pararama molecules homologous to neutrophil degranulation proteins. Several recent studies demonstrate the participation of neutrophil activation in the mechanisms leading to joint damage^8,67^. Proteases such as serine proteases and lysozymes derived from neutrophils are important mediators of inflammation, in addition to playing an important role in the proteolytic activation of cytokines and chemokines^68,69^. These results suggest that, through mimicry of activation by neutrophils, the envenomation leads to an inflammatory response at the joint, which can result in severe local damage and loss of function, which characterize pararamosis.

Finally, the comparison of human-like pararama proteins with the RNA-seq of synovial biopsies from OA and RA patients^33^ revealed seven molecules that are differentially expressed in more than 30% of patients with upregulated proteins. In the context of joint diseases, the role of most of these molecules has not yet been determined. However, their functional mechanisms have been described and can be directly or indirectly related to the installation of pararamosis. On the other hand, some of these molecules are presently being studied and associated with joint diseases.

Of the seven identified proteins are, Agrin (AGRN), an extracellular matrix heparan sulfate proteoglycan (HSPG) with many distinct structural domains^70^, is detected in the cartilage of healthy patients, but its expression progressively decreases in chondrocytes of OA patients^71^, though it is upregulated in the synovial fluid of both OA and RA patients. Aldo–keto reductases (AKR1C2) are responsible for the conversion of prostaglandin (PG) E2 to PGF2α, and play a pro-inflammatory role in OA and RA^72^, such as leukocyte recruitment and stimulation of fibrotic processes in synoviocytes^73^. Cystathionine β-synthase (CBS) is a key enzyme for hydrogen sulphide (H_2_S) production from L-cysteine, as are cystathionine gamma-lyase and 3-mercaptopyruvate sulfurtransferase^74^. H_2_S has been shown to havea significant effect on the regulation of pro-inflammatory cytokines, such as interleukin-6 (IL-6)^75^. It also presents a signal transducer and activator of transcription 3 (STAT3) activity^76–79^. These are important factors involved in the inflammatory process of joint diseases. Gelsolin (GSN) is one of the most important actin-binding proteins, and its decrease in the synovial fluid in RA patients, possibly due to proteolytic degradation, is associated with the local accumulation of actin, causing a worsening of the individual's pathophysiological conditions^80^. Melanotransferrin (MFI2), a protein predominantly found bound to the cell membrane through glycosylphosphatidylinositol, has been implicated in several physiological processes, such as activation of plasminogen and angiogenesis^81^. In the context of OA, the role of MFI2 has not yet been defined. However, the process of angiogenesis and the role of plasmin in the hydrolysis of fibronectin have been described in the pathophysiology of OA^82–84^. Creatine Kinase B (CKB) is associated with cancer and is upregulated in RA. Kelch Domain Containing 4 (KLHDC4) hasn’t yet been associated with any diseases, but is upregulated in OA. These last two, however, are not directly associated with an inflammatory process. In any case, both these proteins, as well as the other five proteins mentioned here, must be further studied in the context of joint diseases.

Here, we theorize on some aspects of the biology of the envenomation by pararama, adding important information on the nature, classes and different groups of protein constituents of the still partially unknown venom of pararama. The transcriptomic and proteomic profiles of pararama showed a mixture of different classes of putative toxins and other proteins that could be related to the clinical manifestations of pararamosis.

The triggering component(s) of the joint disease pararamosis can be undoubtedly found in pararama bristles. On the other hand, the triggering molecule(s) of other joint diseases, such as OA and RA, remain unknown. In our study, a straight correlation between molecules present in the bristles and deregulated in joint diseases, such as OA and RA, could be traced, thus suggesting that the study of pararama could lead to the discovery of molecule(s) involved in other joint diseases.

Using OA and RA databases, some of these pararama proteins were identified as potential key molecules for the development of joint diseases. Developing means to modulate these molecules, especially those related to neutrophils, would eventually be extremely useful for the treatment of different forms of joint diseases.

The limitations of this approach reside in the need for biological validation of the data obtained, which will be provided by studies already on course in our laboratory. Despite these limitations, the highly accurate data generated in this work provides a remarkable diversity of useful information for future studies and advances in the treatment of pararamosis and other joint diseases.

## Supplementary Information

Supplementary Legends.

Supplementary Information 2.

Supplementary Information 3.

Supplementary Information 4.

Supplementary Information 5.

Supplementary Information 6.

Supplementary Information 7.

Supplementary Information 8.

Supplementary Information 9.

Supplementary Information 10.
