# Supplementary material for: Integrative multiomics analysis of Premolis semirufa caterpillar venom in the search for molecules leading to a joint disease
Source: Sci Rep. 2021 Jan 21;11:1995. doi: 10.1038/s41598-020-79769-y (PMC7820220; doi:10.1038/s41598-020-79769-y)
Supplement: Supplementary file 1 — Supplementary Legends. [file 41598_2020_79769_MOESM1_ESM.docx]

**SUPPLEMENTARY INFORMATION**

**Integrative multiomics analysis of *Premolis semirufa* caterpillar venom in the search for molecules leading to a joint disease**

Giselle Pidde^1&*^, Milton Y. Nishiyama-Jr^2&^, Ursula C. de Oliveira^2^, Isadora M. Villas-Boas^1^, Adriana F. Paes-Leme^3^, Inácio L. Junqueira-de-Azevedo^2^, Rafael Marques Porto^4^, Carla C. Squaiella-Baptistão^1^, Denise V. Tambourgi^1*^

^1^Laboratório de Imunoquímica, Instituto Butantan, São Paulo, Brazil

^2^Laboratório Especial de Toxinologia Aplicada, Center of Toxins, Immune-Response and Cell Signaling (CeTICS), Instituto Butantan, São Paulo, Brazil

^3^Laboratório Nacional de Biociências, Centro Nacional de Pesquisa em Energia e Materiais, Campinas, São Paulo, Brazil

^4^Laboratório de Desenvolvimento e Inovação, Instituto Butantan, São Paulo, Brazil

^&^Equally contributed for the article

^*^Corresponding author: denise.tambourgi@butantan.gov.br

**Supplementary Figures**

**Supplementary Figure 1. Schematic workflow.** Schematic representation of combined proteomic and transcriptomic analyses to identify putative proteins in pararama caterpillar bristles. The picture of *P. semirufa* was photographed and kindly provided by co-author Rafael Marques Porto. The figure was drawn using Adobe Photoshop version 2017.1.1 ([www.adobe.com](http://www.adobe.com)/).

# Supplementary Figure 2. Relative FPKM expression levels of putative toxins. Classification of putative toxins in the pararama (A) integument and (B) the corresponding ones in the bristles. The figure was drawn using Microsoft Excel version 1.5 (www.microsoft.com/).

**Supplementary Figure 3. Multiple sequence alignment and phylogenetic analysis.** (**A**) Alignment of pararama serine protease domains with serine proteases from other lepidopterans. The conserved catalytic triads for serine protease activities are indicated by #. Identical residues (>75%) are highlighted in black while similar amino acids are highlighted in gray. Multiple sequence alignment of molecules transcripts was performed using ClustalWS method. (**B**) Phylogenetic relationship between pararama serine proteases and other homologous serine protease sequences. Maximum Likelihood phylogenetic analysis of putative serine proteases from pararama*.* We identified 3 clans, snake-like serine protease (2 proteins), serine protease (3 proteins) and trypsin like (3 proteins). The figure was drawn using FigTree version 1.4.0 (http://tree.bio.ed.ac.uk/software/figtree/).

**Supplementary Figure 4. Homologous proteins and statistical significance.** Comparison of pararama bristles extract proteins with human proteins, using BlastX alignment tool, resulted in 129 proteins with a high level of similarity. The boxplots present the data in %Identity, %Positive and %Qcoverage. The figure was generated in R-statistics version 3.63, using the package ggplot2 version 3.2.1 (https://ggplot2.tidyverse.org/).

**Supplementary Figure 5. Significant interaction network analysis.** The Indirect MetaCore interaction algorithm was used to generate the interaction network. Several signaling pathways are possibly triggered by proteins from the pararama bristles extract, which can lead to the activation of genes/proteins involved in the regulation of the inflammatory response. The interaction network comprised 76 genes that contained 25 significant genes (red circle) identified in this study. The figure was drawn using MetaCore version 6.27 (<https://portal.genego.com>/).

**Supplementary Tables**

**Supplementary Table 1.** Protein identification list performed using LC-MS/MS analysis.

**Supplementary Table 2**. Putative toxins identified in the transcriptome analysis. The highlighted proteins were also identified in the proteome analysis.

**Supplementary Table 3.** Comparison of 129 homolog genes between *H. sapiens* and *P. semirufa*, represented by the identified domains in the proteins for each species by Batch CD-Search Tool against the NCBI-curated domains from Conserved Domain Database (CDD), Pfam, SMART, COG, PRK, and TIGRFAM. There were included in the table the BlastX alignment statistics for the %Identity, %Positives, %Query coverage and E-value.

**Supplementary Table 4.** Summarization for the number of identified domains in each specie and the percentage of common domains for each species based on the Conserved Domains Search Tool against the databases CDD, Pfam, SMART, COG, PRK, and TIGRFAM.
