# Supplementary figures and images for: Integrative multiomics analysis of Premolis semirufa caterpillar venom in the search for molecules leading to a joint disease
Source: Sci Rep. 2021 Jan 21;11:1995. doi: 10.1038/s41598-020-79769-y (PMC7820220; doi:10.1038/s41598-020-79769-y)

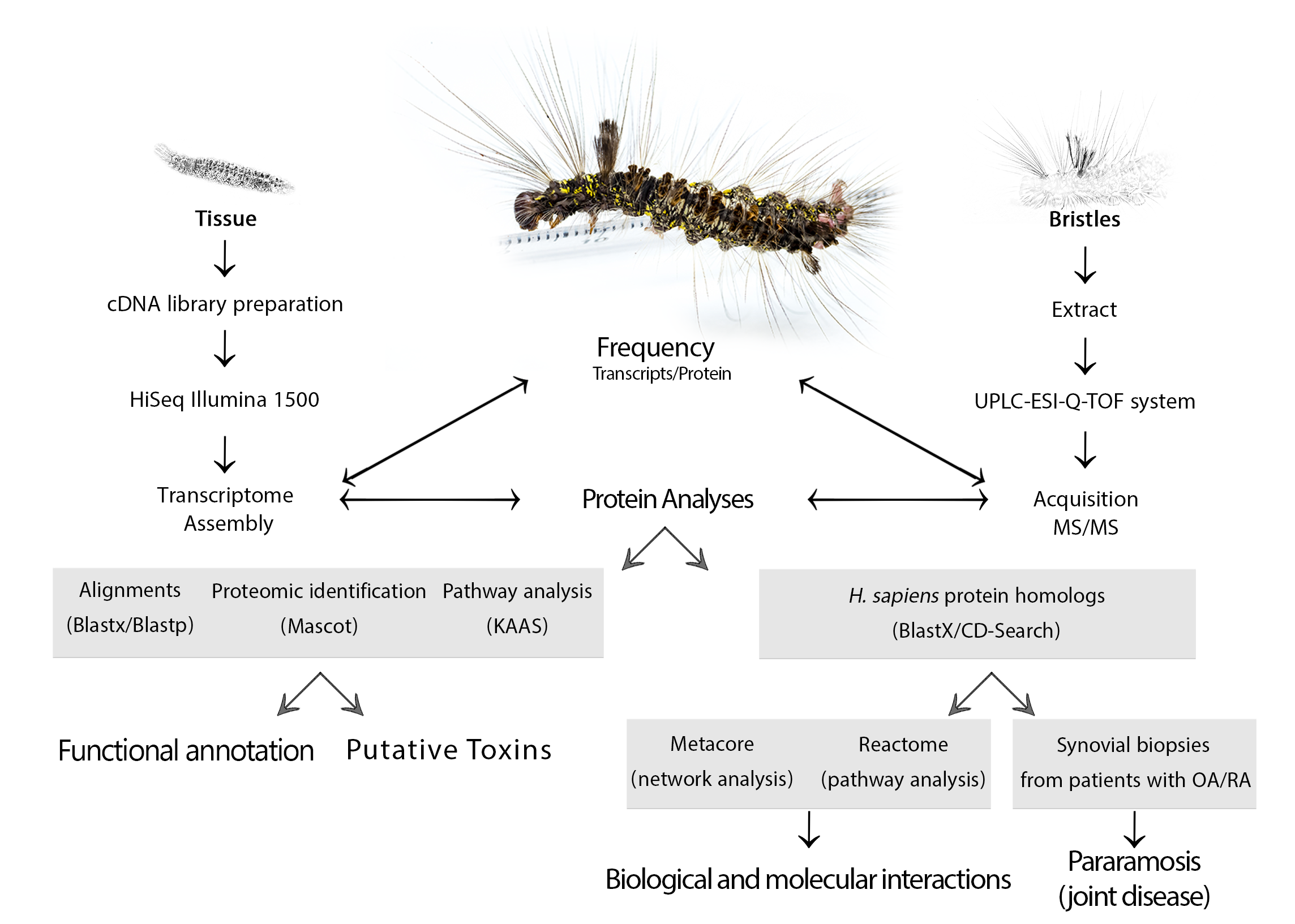

Supplement: Supplementary file 2 — Supplementary Information 2. [file 41598_2020_79769_MOESM2_ESM.tif]

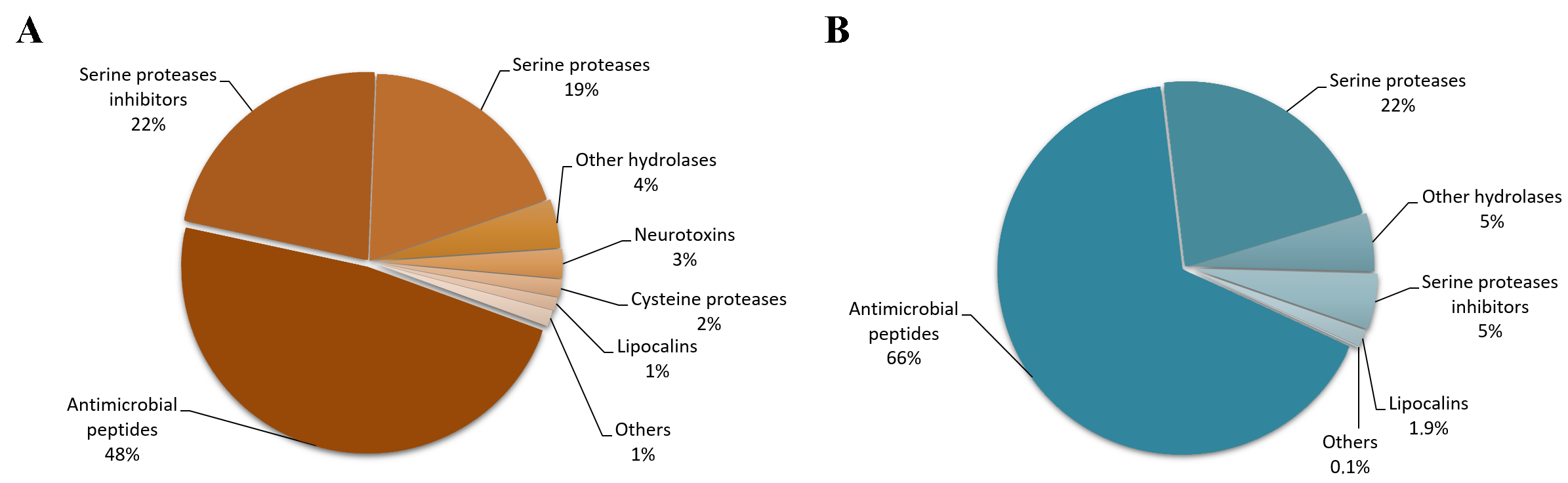

Supplement: Supplementary file 3 — Supplementary Information 3. [file 41598_2020_79769_MOESM3_ESM.tif]

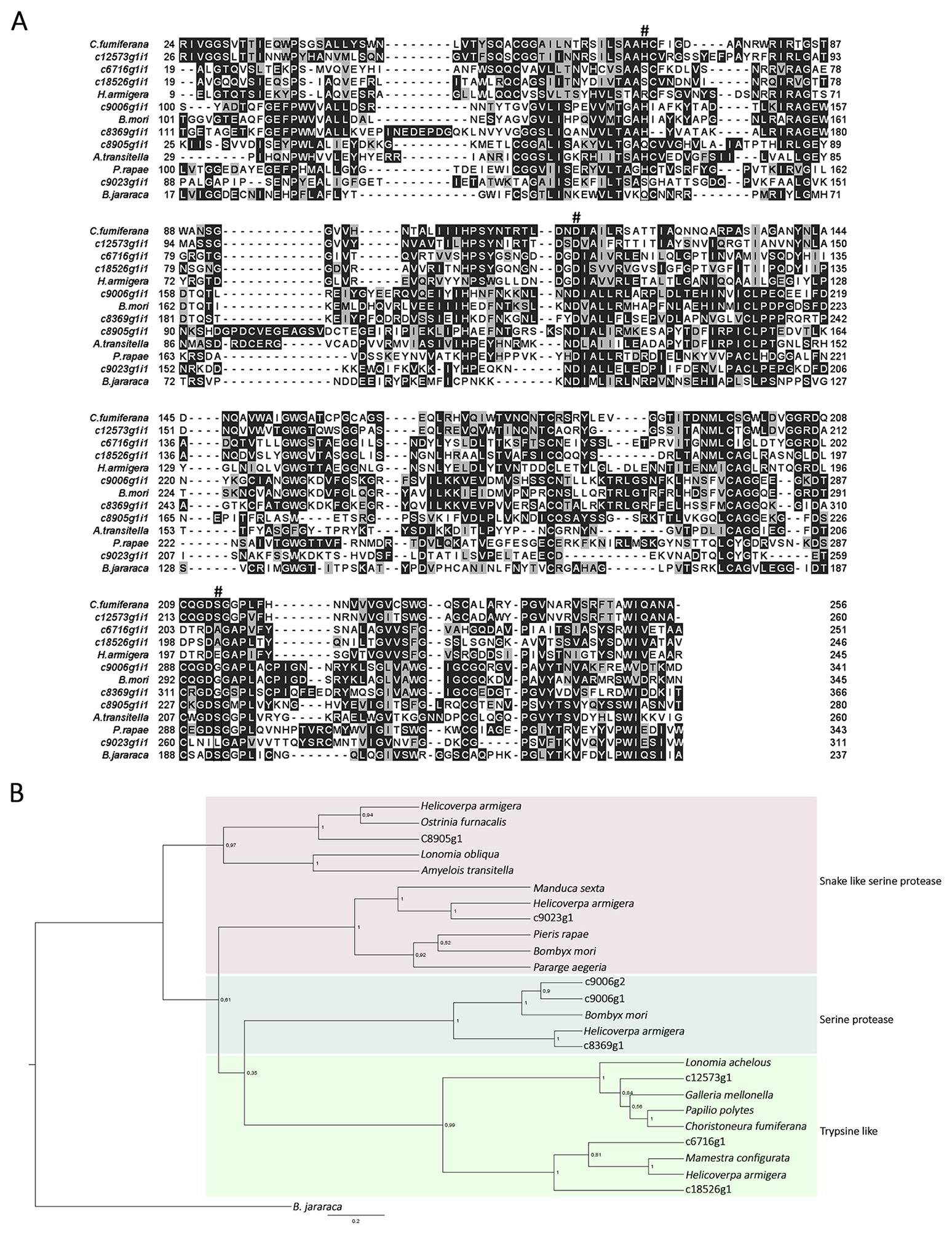

Supplement: Supplementary file 4 — Supplementary Information 4. [file 41598_2020_79769_MOESM4_ESM.tif]

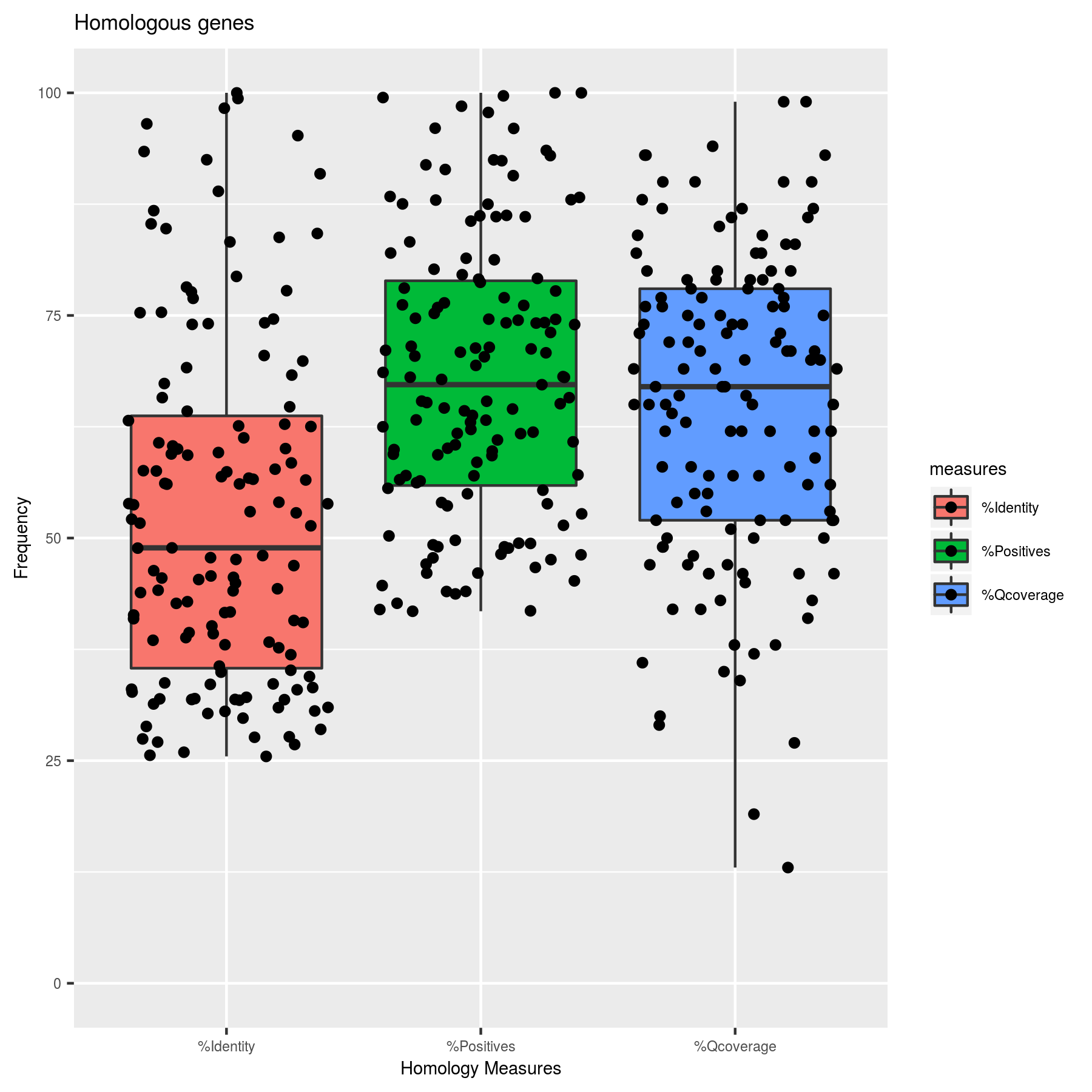

Supplement: Supplementary file 5 — Supplementary Information 5. [file 41598_2020_79769_MOESM5_ESM.tiff]

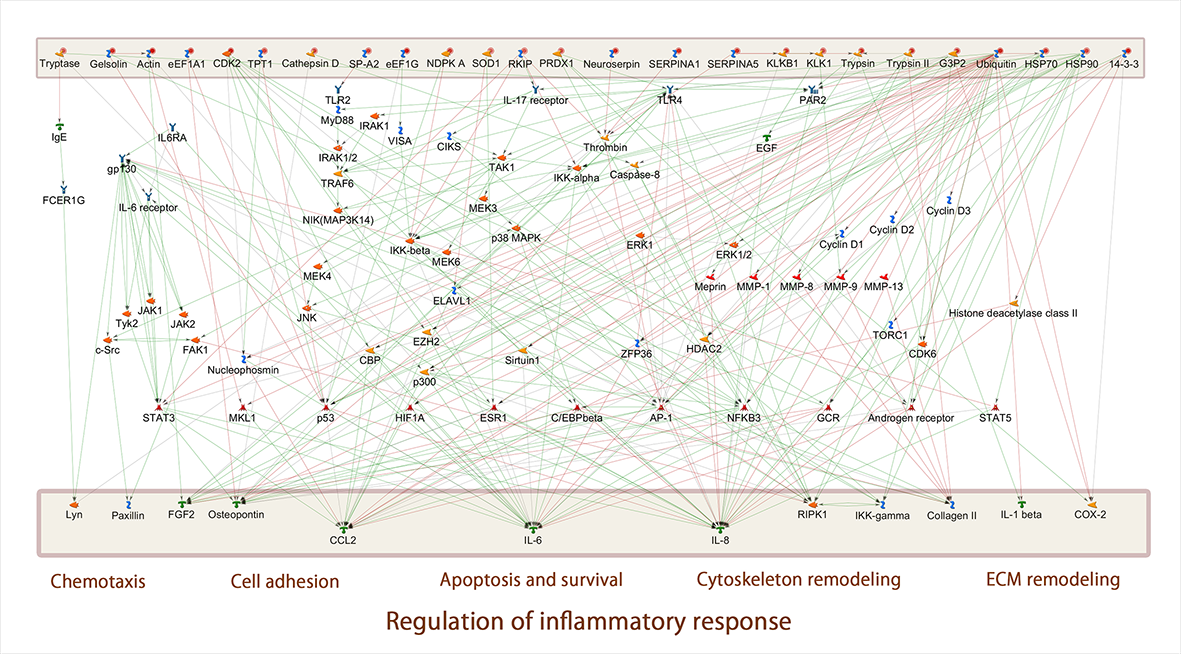

Supplement: Supplementary file 6 — Supplementary Information 6. [file 41598_2020_79769_MOESM6_ESM.tif]
